# Tailoring relaxation dynamics and mechanical memory of crumpled materials by friction and ductility

**DOI:** 10.1039/c8sm01951g

**Published:** 2019-01-15

**Authors:** Eric van Bruggen, Erik van der Linden, Mehdi Habibi

**Affiliations:** Laboratory of Physics and Physical Chemistry of Foods, Wageningen University Wageningen The Netherlands mehdi.habibi@wur.nl

## Abstract

Crumpled sheets show slow mechanical relaxation and long lasting memory of previous mechanical states. By using uniaxial compression tests, the role of friction and ductility on the stress relaxation dynamics of crumpled systems is investigated. We find a material dependent relaxation constant that can be tuned by changing ductility and adhesive properties of the sheet. After a two-step compression protocol, nonmonotonic aging is reported for polymeric, elastomeric and metal sheets, with relaxation dynamics that are dependent on the material's properties. These findings can contribute to tailoring and programming of crumpled materials to get desirable mechanical properties.

## Abstract 2

Crumpled sheets show slow mechanical relaxation and long lasting memory of previous mechanical states.

## Graphical abstract

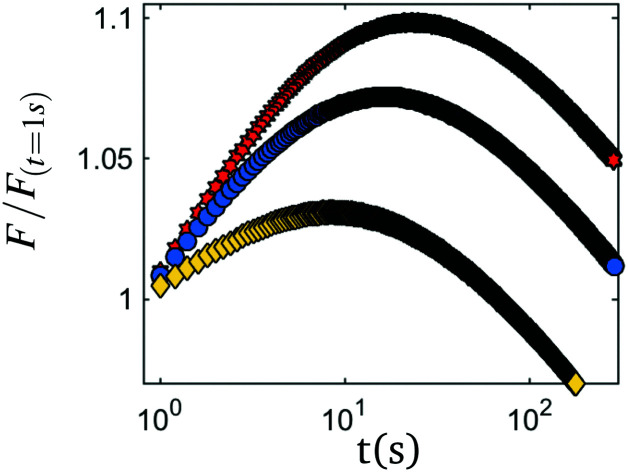

## 1 Introduction

Crumpled structures are found in many areas of research as well as in a large range of length scales: from DNA packed in viral capsids^1–3^ and crumpled graphene^4^ to crumpled paper balls, car wrecks, or even the geological stratum.^5^ Crumpled systems (CSs) can combine low density structures with surprising mechanical strength and the ability to absorb mechanical energy.^6–9^ This combination of properties opens doors to use CSs for a variety of applications such as shock absorbers or light weight sandwich panels.^10,11^ However, in order to rationally design disordered materials, a thorough physical understanding of their unique features is needed. One remarkable physical property observed in CSs is their slow mechanical relaxation and their ability to carry long lasting memory of previous mechanical states. Research on conductors, glasses and polymers^12^ suggests that slow relaxation dynamics are a generic feature of amorphous materials and ubiquitous. In spite of a significant amount of literature on crumpled materials, a lot of questions about the mechanisms that govern slow stress relaxation are still unanswered. Hitherto, several theoretical models have been suggested to predict force recovery dynamics in CS. The two most popular descriptions being the Kohlrausch function (‘stretched exponential’):^13–15^

1 *F*(*t*) = *a*_1_·(e^−*t*/*τ*^)^*b*_1_^

and the logarithmic decay function:^8,16–18^

2 *F*(*t*) = *a*_2_ + *b*_2_ log(*t*/*τ*)

Both models are capable of describing a broad range of behaviours in the development of force over time *F*(*t*) over many orders of magnitude^19,20^ with two free variables (*a*_*i*_ and *b*_*i*_) and a characteristic time scale *τ* (*t*/*τ* further denoted as t*). The stretched exponential function has been used to model luminescence decay of colloidal quantum dots,^21^ remnant magnetization in spin glasses^22^ or structural relaxation in glasses and polymers,^23^ while the logarithmic function has been widely applied to model slow processes such as decay of current in superconductors,^24^ conductance relaxation in electron glasses^25^ and mechanical relaxation of plant roots.^12,26^

Extensive effort has been put into developing microscopic models that are compatible with eqn (1) or (2). For example Palmer *et al.*^27^ show how a stretched exponential function can naturally arise from hierarchically constrained dynamics. Amir *et al.*,^12^ provide an interesting discussion on how logarithmic relaxation might be explained from a *P*(*λ*) ∼ 1/*λ* probability distribution (*P*) of relaxation rates (*λ*) that can emerge from various random multiplicative processes. More discussion on the topic can be found in ref. 28 and 29. However, most models still lack a clear physical basis that links the material's micro-structure to the observed long time scale relaxations.^23^

A remarkable observation is that despite the similarities in stress relaxation for various crumpled systems, the stretched exponential model is preferred to describe crumpled stress relaxation in aluminum systems,^13–15^ whereas the logarithmic model seems to be more capable of fitting mechanical relaxation of crumpled paper and Mylar.^8,16–18^

The fact that material properties have a profound effect on the mechanical response of crumpled structures has already been convincingly shown. Many numerical^30,31^ and experimental^6,10,32,33^ studies have been dealing with the effect of elastoplasticity in the crumpling process. Matan *et al.*^8^ showed that the force needed to crumple a sheet (*F*) obeys a power law that depends on the size (*D*) of the crumpled object (*F* ∼ *D*^−*β*^), where the exponent *β* is material dependent. This was further quantified by Habibi *et al.*^10^ who showed that *β* is plasticity dependent and can be related to a dimensionless parameter called a foldability index, which is a measure for the ductility in the material. The effect of friction, adhesion and other surface interactions is an often ignored part of the crumpling process.^13,17,34^ Structural relaxation can also be influenced by the material properties and friction, but to what extent they can determine a crumpled material's relaxation behaviour is an open question.

In this paper, we present a comparative study where we look into a set of materials with different ductilities and friction coefficients and compare their relaxation behaviour after uniaxial compression. Moreover, we make use of a compaction protocol of Lahini *et al.*^18^ to further characterize and compare the material's aging behaviour. Our findings can help elucidate the origins of slow stress relaxation in disordered crumpled structures and will provide insight in rational design of future crumpled materials.

## 2 Experimental procedure

### 2.1 Sample preparation

A variety of widely available sheet materials was used. Regular printing paper, BOPP (biaxially oriented polypropylene) used as transparent wrapping sheet, rubber, aluminum and brass sheets offered an economic means for studying crumpling phenomena while at the same time representing a wide range in elastoplastic response and frictional coefficients. In addition to using different materials, elastomers were powdered with a very fine corn starch flour, creating a minimal layer of starch particles as to reduce friction and adhesion without altering its bulk properties.

### 2.2 Friction measurement

**Fig. 1:**
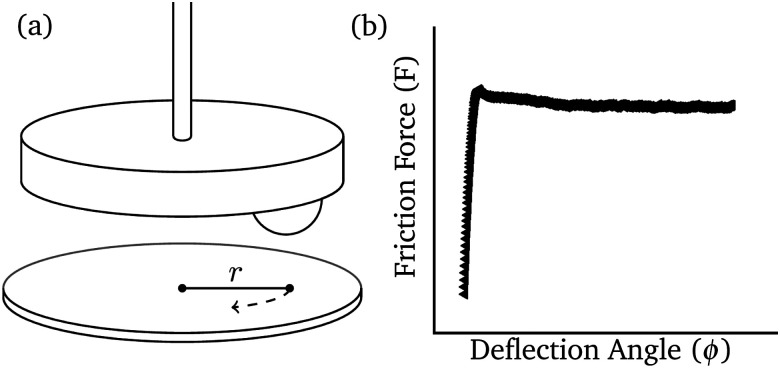
(a) Schematic drawing of friction measurement setup using a rheometer. Material is attached both onto the upper sphere and onto the bottom plate. The sphere is lowered onto the plate till a certain *F*_N_ is reached. As the probe rotates, torque *τ*, angle of deflection *ϕ* and normal force *F*_N_ are measured, from which the friction coefficient can be calculated. (b) A typical friction curve as measured with the rheometer. The static friction force is the peak friction value measured before the slip. The dynamic friction force is the value of the friction force as it reaches a plateau.

To give a measure for both static and dynamic friction coefficients of our sheet materials, a standard rheometer (Anton Paar MCR 302) was transformed into a tribometer with a few simple adjustments.^35^ First a glass sphere was attached to a plate. Both glass sphere and bottom plate were covered carefully with the sheet material (Fig. 1a). To start a measurement, the probe is brought into contact by lowering the measuring tool. The normal force is controlled by vertical displacement of the glass sphere. The probe follows a circular trajectory on the bottom plate. Torque *τ*, angle of deflection *ϕ* and normal force *F*_N_ are measured during rotation. Dividing the torque by length of the arm *r* ∼ 6 mm gives us a frictional force *F* = *τ*/*r*. Following Amonton's second law of friction, the frictional force is proportional to the magnitude of the normal force. A frictional coefficient (*μ*) can be determined by dividing frictional force by the normal force *F*_f_/*F*_N_ = *μ*. At small deformations, a peak in frictional force can be observed, that corresponds to the static friction coefficient *μ*_s_. At larger deformations frictional force reaches a plateau where we find the kinetic friction coefficient *μ*_k_ (Fig. 1b).

### 2.3 Stress relaxation procedure

All samples were subjected to uniaxial compression tests in a closed die on a TTC TA.XT *plus* texture analyser. Crumpled samples were prepared by confining a sheet of roughly A4 size by hand in a hollow plexiglass cylinder of diameter and height of 50 mm. Sheets were compressed by a cylindrical plexiglass probe under a constant strain rate of 2 mm s^−1^ until a pre-set force was reached. The force exerted on the probe was measured over time under constant strain. For every material, a number of samples were taken, varying the initial compaction force within range *F* = 1–200 N.

### 2.4 Two-step compression protocol

**Fig. 2:**
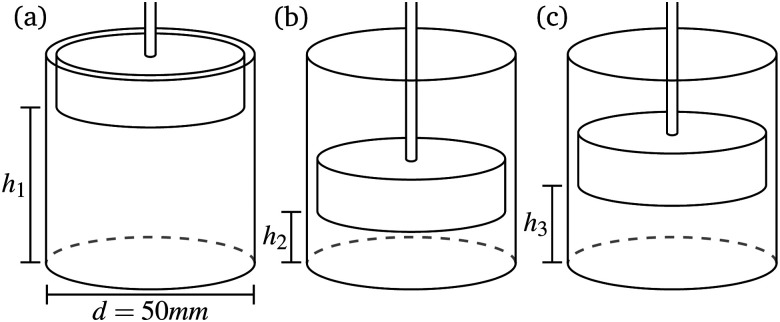
Schematic representation of compaction protocol for two step compaction protocol with *h*_1_ < *h*_3_ < *h*_2_ – height *h*_*i*_ corresponds to force *F*_*i*_.

To look further into the fundamentals of stress relaxation, a procedure, proposed by Lahini *et al.*^18^ was followed, *i.e.* a two-step compression protocol. After initial compression from height *h*_1_ to *h*_2_ (with force *F*_1_ to *F*_2_), strain was kept constant for a specific waiting time *t*_w_ after which the probe was lifted to *h*_3_ with a strain rate of 5 mm s^−1^ towards a new force *F*_3_ for which *F*_1_ < *F*_3_ < *F*_2_ (Fig. 2). The normal force was continuously measured over time. *h*_1_ was set in a way that *F*_1_ ∼ 0.1 N was obtained, while *F*_2_ varied in between 20–50 N and *F*_3_ between 1–45 N.

## 3 Results and discussion

**Fig. 3:**
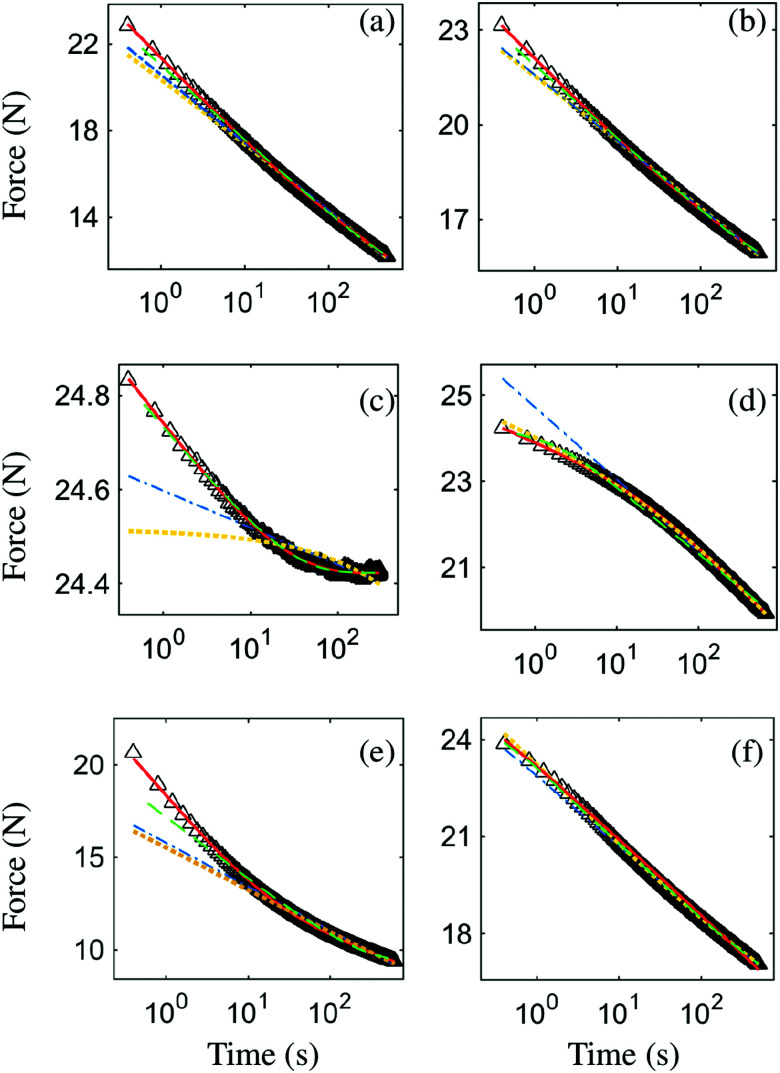
Stress relaxation over time – examples shown for stress relaxation with initial compaction of *F* ∼ 25 N. Stretched exponential *F* = *a*·e^t*^b^^ (yellow, dotted) logarithmic with *F* = *a* + *b* log(t*) (blue, dash-dot), exponential integral function *F* = *a* + *b*[*E*_1_(*λ*_min_*t*) − *E*_1_(*λ*_max_*t*)] (green, dashed) and double log-function *F* = *a* + *b* log(t*) + *c* log(t* − *t*_0_*) (red, solid) are fitted for relaxation data of (a) BOPP, (b) paper, (c) brass, (d) aluminium, (e) rubber and (f) rubber coated with fine starch powder.

Typical stress relaxation curves for the tested materials are shown in Fig. 3 that show relaxation after initial compaction at *F* ∼ 25 N. All studied samples show slow logarithmic-like stress relaxation with a steep decrease in magnitude of stress for short times <20 s and a slow but persistent decrease in the magnitude of stress for greater time scales. Relaxation data were fitted with eqn (1) and (2). All data sets show (at least slight) non-stochastic deviations from both the logarithmic and exponential model. Note that for aluminium (Fig. 3d) first a slower relaxation regime is observed, followed by a faster (logarithmic) relaxation, while for the BOPP, printing paper and brass (Fig. 3a–c) relaxation seems to slightly slow down for larger time scales. In line with published literature, it can be seen that relaxation of crumpled aluminium is very well described by a stretched exponential model, while paper and BOPP show a trend that closely resembles logarithmic decay.

We can expand the logarithmic model by including physical cutoff rates *λ*_max_ and *λ*_min_. Eqn (2), was based on the assumption that during our experiments time scales far from the inverse of the extreme relaxation rates are considered: 1/*λ*_max_ ≪ *t* ≪ 1/*λ*_min_. If this is not the case, we can revert to the more general equation:^36^

3 *F*(*t*) = *F*_0_[*E*_1_(*λ*_min_*t*) − *E*_1_(*λ*_max_*t*)]

That is the sum of two exponential integrals *E*_1_ which enables us to incorporate a minimum and maximum rate of relaxation that can account for a plateau at very small or very large time scales. In Fig. 3 fits to eqn (3) are plotted. Especially for the brass sample (Fig. 3c), eqn (3) is very able to describe the observed relaxation behaviour, indicating that the examined time scales are not far from 1/*λ*_min_ and thus responsible for the flattening of the curve near *t* ∼ 10^2^. For the aluminium sample flattening near *t* ∼ 10^0^ occurs (Fig. 3c), indicating that examined time scales are not far from 1/*λ*_min_.

However, applying finite cutoff rates cannot account for the bended shape that is apparent for the rubber sheets (Fig. 3e) and to lesser extent can also be observed for both BOPP and paper samples (Fig. 3a and b). Instead, these apparent curved lines bare remarkable similarities to the results obtained by Lahini *et al.* for elastic foams^18^*i.e.* two logarithmic regimes can be distinguished and in a similar fashion, we can use a superposed logarithm to fit our data:

4 *F* = *a* + *b* log(t*) + *c* log(t* − *t*_0_*)

Eqn (4) provides a solid fit to all our data and plots of the residuals show a stochastic spread in a large time window. Non-stochastic deviation can still be seen at short time scales *t* < 20 s which we attribute to the finite strain rate during compaction, the associated premature relaxation and the earlier discussed finite maximum cutoff rate.

### 3.1 Relaxation rate

**Fig. 4:**
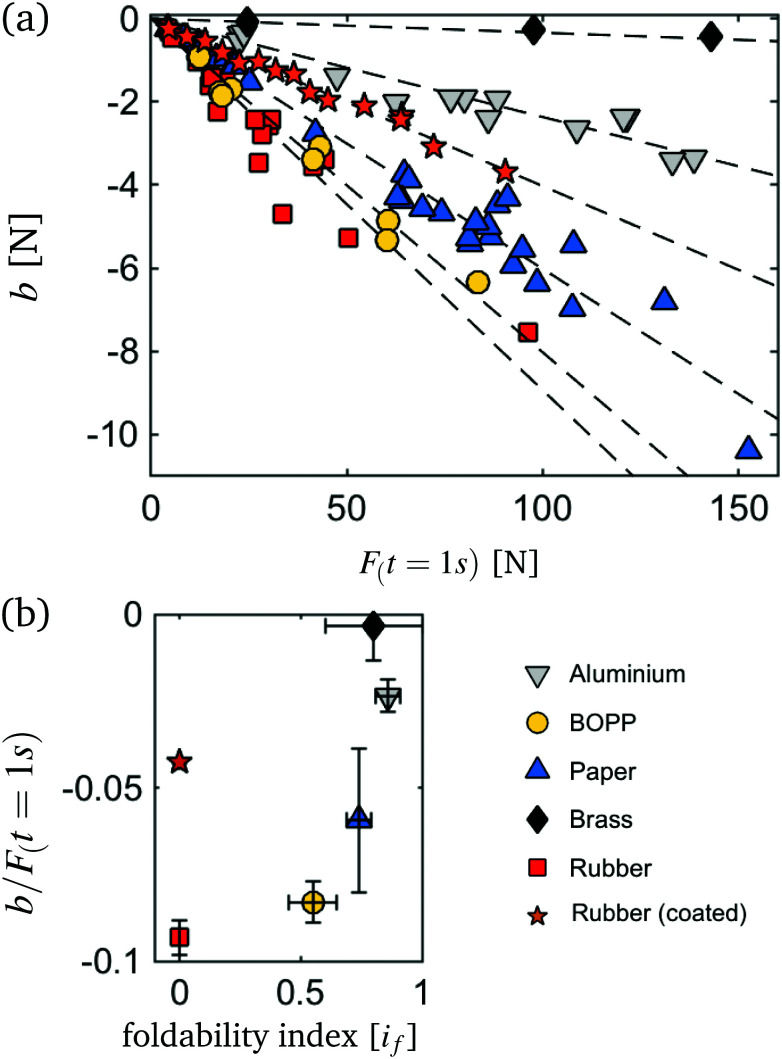
(a) Material dependent relaxation rates, with rate *b* from equation *F* = *a* + *b* log(t*) + *c* log(t* + *t*_0_*). Dashed lines are least square fits to the data forced through the origin. Increased initial compaction force *F*_(*t*=1s)_, leads to an increased relaxation rate. (b) The material parameter ‘*b*/*F*_(*t*=1s)_’ is plotted against a dimensionless foldability index (results measured using the procedure of Habibi *et al.*).^10^

The applicability of eqn (4) to all our tested samples, allows us to do an inter material comparison of its plotting parameters set out against a range of initial compaction forces *F*_(*t*=1s)_ (the force measured 1 second after compaction) to which our samples were subjected (see Fig. 4a).

We report a linear dependence of fitting parameter *b* on the initial compaction force *F*_(*t*=1s)_. Notice that *b*/*F*_(*t*=1s)_ is constant for all initial packing forces and protocol independent and thus reflects an intrinsic material property. Our results are comparable to the findings for relaxation of a single fold in polymeric sheets by Thiria *et al.*^37^ who reported an intrinsic material constant in the single folding of sheets, but to our knowledge, it is the first time such a constant is proposed for crumpled materials.

To systematically investigate how the material dependent relaxation constant for CSs *b*/*F*_(*t*=1s)_ is affected by the sheet's material properties, a dimensionless foldability index *i*_f_ is employed. *i*_f_ introduced by Habibi *et al.* gives a measure for the material's ductility or behaviour beyond yielding.^10^ In Fig. 4b constant *b*/*F*_(*t*=1s)_ is plotted as a function of the foldability index. For most tested materials, an increase in ductility is accompanied by a higher relaxation constant. However, this observation fails for the starch powdered rubber material that has an *i*_f_ comparable to the untreated rubber sheet, but a distinctly different response, indicating that surface phenomena are at play.

### 3.2 Effect of friction

Using the setup of Fig. 1, friction coefficients for all sheet materials were measured. Results are shown in Table 1. The determination of the friction coefficient for rubber was complicated by stick-slip^38^ and adhesive phenomena.^39^ For adhesive materials, frictional force depends nonlinearly on the applied load and the restriction *F*_f_ = 0 at *F*_N_ = 0 is not valid,^40^ where *F*_f_ and *F*_N_ = 0 are friction and normal forces respectively. Since there is no linear dependence, the friction coefficient is ill-defined. Nevertheless, we included the calculated value in Table 1 as it provides insight in the rubber's friction-like behaviour.

**Table 1:** Friction coefficients for material samples

Sheet material	Dynamic friction *μ*_k_	Static friction *μ*_s_
Aluminium	0.17 ± 0.01	0.21 ± 0.03
Paper	0.29 ± 0.02	0.37 ± 0.03
Rubber^a^	2.71 ± 0.99	3.36 ± 1.65
Rubber (coated)	0.36 ± 0.04	0.41 ± 0.04
BOPP	0.50 ± 0.03	0.53 ± 0.05

a No ‘true’ friction coefficient (see discussion).

After a coating of corn starch flour is applied to the rubber elastomer, stick-slip phenomena are no longer observed. Moreover, the apparent friction coefficient is considerably lower, confirming that indeed, addition of starch is an effective way to reduce adhesion and lower the friction coefficient, thus allowing us to control the surface properties of our rubber material.

Returning to the simple relaxation experiment: in Fig. 3 we observed that addition of the very fine corn starch powder has a profound impact on the stress relaxation of rubber. Reduction of friction through addition of corn starch changes the shape of the relaxation data from a bend line in the semi-log plot to a straight line that can be very well described by a single logarithmic model.

A double logarithm can be used to describe the bend relaxation curves, reminiscent of the shapes obtained by Lahini *et al.* for relaxation of the polymeric foams. That crumpled systems showing foam-like properties, has already been convincingly pleaded by Bouaziz *et al.*,^32^ but relaxation in polymeric foams has shown markedly different behaviour from crumpled polymeric sheets.^18^ Moreover, it has been argued that friction has only a small effect on the crumpling process.^14,30,32^ However, in present literature only materials with friction coefficients smaller than one and frictional forces that vanish near zero load have been considered. The examined rubber sample now gives us a means to investigate samples with higher friction coefficients and a finite friction near zero load due to adhesive contacts. One can hypothesize that due to these adhesive contacts, inter-layer movements during compaction and relaxation are reduced to a minimum, or even are completely absent resulting in the foam-like relaxation behaviour of the adhesive sheet.

Furthermore, under an equal compaction force, untreated rubber has a considerably faster relaxation rate than the starch powdered samples (Fig. 4a). A similar observation was made by Cottrino *et al.*:^14^ applying a higher strain rate resulted in a faster relaxation rate, and it has been explained by the inability of the internal micro-structure to properly rearrange itself.

In the same train of thought, we can assume that due to increased surface interactions, crumpled structures are not able to rearrange properly during compaction. This leads to an increased storage of energy in the form of ridges and vertices during the crumpling process.^41^ Consequently, after compaction a system high in friction is further away from its equilibrium state than its frictionless counterpart, which can explain the larger relaxation rate of the adhesive rubber sample at similar initial compaction forces (Fig. 4).

### 3.3 Non monotonic aging

**Fig. 5:**
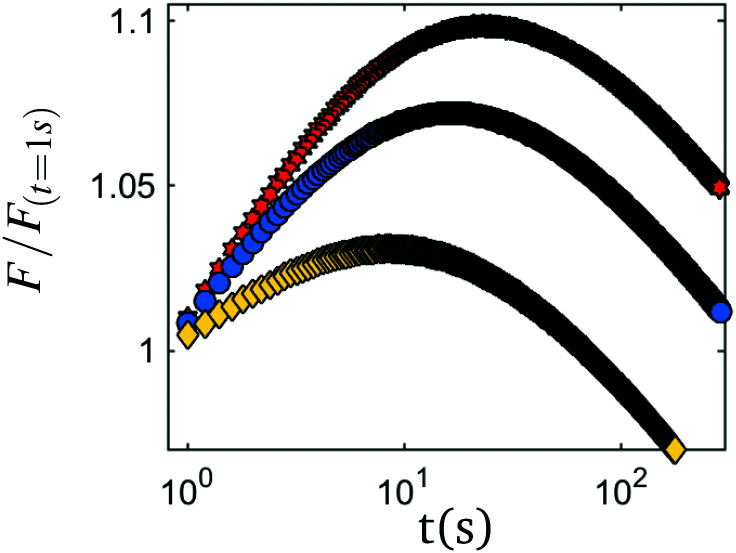
Examples of nonmonotonic stress relaxation – depending on the compaction protocol, peak time and peak force change. Shown are coated rubber samples with *F*_1_ = 0.1 N, *F*_2_ = 40 N, while *F*_3_ = 12 N (red hexagons), *F*_3_ = 18 N (blue circles) and *F*_3_ = 22 N (yellow diamonds).

In Fig. 5 stress evolution in time after the two step compression protocol is plotted. After decompression from *F*_2_ to *F*_3_, non-monotonic stress relaxation is apparent for all tested materials: the normal force shows a steep increase for the first seconds to minutes, then reaches a well-defined peak at a time *t*_p_, and after, resumes slow decay. This means that before and after the peak, sets of data-points exist for which the strain, force and all other macroscopic observable are identical, however the direction of the force evolution is qualitatively different, indicating that force evolution cannot be predicted based on these observable parameters on its own. Instead, one needs to take into account the history of the system or deal with the system's ‘memory’.

The occurrence of non-monotonic relaxation after decompaction can be explained as a consequence of the non-equilibrium states of the system during relaxation.^18^ Starting point is the assumption that the system consists of an ensemble of relaxation modes, covering a wide range of relaxation rates that is at rest in an equilibrium state *E*_1_ before compaction (at *F*_1_). By moving the piston to *F*_2_, a new equilibrium state *E*_2_ is forced upon the system, to which all relaxation modes move towards while contributing to the total force opposite of the compression direction. Over time, this force is decreasing as more and more relaxation modes arrive in close vicinity of *E*_2_. By introducing a decompression step to *F*_3_, we force a third equilibrium state *E*_3_ upon the system for which *E*_1_ > *E*_3_ > *E*_2_. We now see that the fast relaxation modes which were already in close vicinity to *E*_2_ and thus >*E*_3_, will now possess an sign compared to that of the slow relaxation modes that were still close to the original equilibrium state *E*_1_ and for which >*E*_3_ is valid. In the short time towards the peak, contributions of the fast relaxation modes are prevalent, thus leading to the observed peak in normal force.

**Fig. 6:**
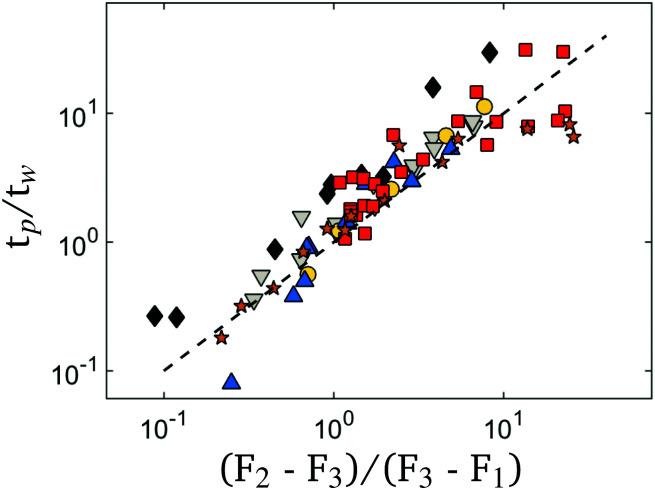
Ratio *t*_p_/*t*_w_ against the ratio of forces (*F*_2_ − *F*_3_)/(*F*_3_ − *F*_1_) for metal, polymeric and elastomeric crumpled systems. Dashed line with (*t*_p_/*t*_w_)/[(*F*_2_ − *F*_3_)/(*F*_3_ − *F*_1_)] = 1 provides a reasonable fit for all data.

If the waiting time *t*_w_ at *F*_2_ is varied, while other conditions are kept constant, it is found that peak time *t*_p_ depends linearly with *t*_w_ and a slope *t*_p_/*t*_w_ can be extracted. The steepness of slope *t*_p_/*t*_w_ in its turn is dependent on the compaction protocol. If we assume that the compaction force is proportional to an intrinsic equilibrium state of the crumpled system, it can be derived that *t*_p_/*t*_w_ = (*F*_2_ − *F*_3_)/(*F*_3_ − *F*_1_).^18^ In Fig. 6, ratio *t*_p_/*t*_w_ is plotted against the force ratio (*F*_2_ − *F*_3_)/(*F*_3_ − *F*_1_). Indeed we find that [(*F*_2_ − *F*_3_)/(*F*_3_ − *F*_1_)]/[*t*_p_/*t*_w_] ≈ 1 independent of the used material and without the need for any fitting parameters. This implies that non-monotonic aging is a generic feature of crumpled systems, governed by universal relaxation dynamics.

**Fig. 7:**
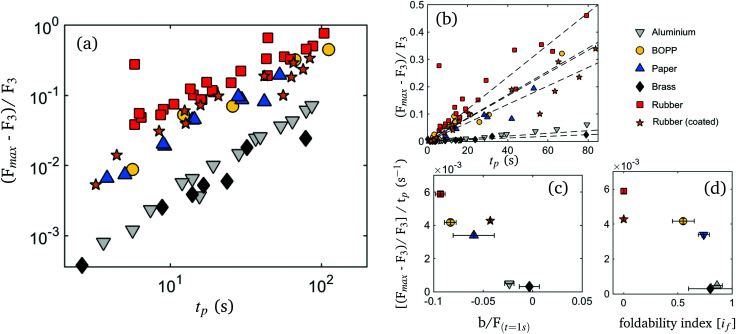
(a) Normalized peak force plotted against the peak time *t*_p_ – the plastic aluminium and brass behave markedly different from the polymeric compounds. (b) Same as in (a) on linear scale. Dashed lines represent fits by robust bi-square fitting, restricted to the origin. (c) Slopes of (b) plotted against the material dependent relaxation *b*/*F*_(*t*=1s)_.

In order to compare non-monotonic dynamics of different experiments, measured force is normalized by *F*_3_ (at *t* = 1 s after decompression). Plotting the normalized forces at *t*_p_ (*F*_max_) of different compaction protocols, shows that *F*_max_ increases linearly with peak time *t*_p_ (Fig. 7). Comparison of the different sheet materials, reveals a material dependent slope in units of s^−1^. The order of slopes bares remarkable similarity to that of the relaxation constant *b*/*F*_(*t*=1s)_, implying that the same mechanisms are at play (Fig. 7c). In Fig. 7d obtained slopes are plotted as a function of foldability index and indeed a correlation between ductility and the maximum normalized force recovery is apparent. This fits the theory that plastic sheets are less able to story energy during compaction due to yielding and plastic flow. Moreover, we find that the untreated rubber sample shows a weaker normalized force recovery than the starch coated sample, indicating that friction and adhesion again are at play. We explain our finding with the idea that adhesive samples are capable of storing more elastic energy during compaction than frictionless samples, that can be released at time scales similar to *t*_p_.

Our findings open windows for the controlled design of crumpled systems: by changing the adhesive properties of the sheet's surface, the crumpled system's stress response can effectively be altered, while maintaining the bulk elastoplastic response. Furthermore, by selecting materials based on ductility, we can tailor their nonmonotonic aging behaviour and mechanical memory.

## 4 Conclusions

In conclusion, we investigated the role of material properties on relaxation dynamics by comparing relaxation curves of multiple materials and proposed a double logarithm to model stress relaxation in CSs. We found that relaxation rates are not only dependent on material's elastoplastic properties, but also rely on friction and adhesion. This was further explored by using a two-step compaction protocol, that allowed us to probe deeper into the material's relaxation behaviour. For all materials we found nonmonotonic aging, with the appearance of a peak force at a certain peak time. We confirmed that [(*F*_2_ − *F*_3_)/(*F*_3_ − *F*_1_)]/[*t*_p_/*t*_w_] ≈ 1 and thus the notion that nonmonotonic aging is a generic feature of crumpled systems whose timescales are solely governed by the compaction protocol. However, the normalized height of the nonmonotonic aging peak, was found to depend linearly on the time at which it arose with a slope that revealed a material property that seemed to be correlated with the material dependent relaxation constant. With this paper, we contribute to an increased understanding of crumpled systems that can help in further tailoring and programming of these disordered materials.

## Conflicts of interest

There are no conflicts of interest to declare.

## Supplementary Material
